# Tubercular Retropharyngeal Abscess With Pott’s Disease in an Elderly Male Patient

**DOI:** 10.7759/cureus.8256

**Published:** 2020-05-23

**Authors:** Kiren Thomas, Manish Gupta, Saurabh Gaba, Monica Gupta

**Affiliations:** 1 Otolaryngology, Maharishi Markandeshwar Institute of Medical Sciences and Research, Ambala, IND; 2 Otorhinolaryngology, Maharishi Markandeshwar Institute of Medical Sciences and Research, Ambala, IND; 3 General Medicine, Government Medical College and Hospital, Chandigarh, IND

**Keywords:** retropharyngeal abscess, adults, tuberculosis, surgical drainage, cervical spine

## Abstract

Retropharyngeal abscess (RPA) is a life-threatening emergency due to its potential to cause airway compression. It is rare in the elderly and occurs mostly in immunocompromised patients, or as a complication of instrumentation. We are reporting the case of a 70-year-old male who presented with sudden onset breathing difficulty with a history of dysphagia for three months. The clinical examination revealed a bulge in the posterior pharyngeal wall. A lateral-view radiograph of neck revealed retropharyngeal soft tissue density with carious spine. The patient was successfully treated by trans-oral incision and drainage of the abscess under local anesthesia. Diagnosis of tuberculosis was confirmed by positive acid-fast staining and cartridge-based nucleic acid amplification test (CBNAAT). The patient improved significantly following the initiation of anti-tubercular therapy.

## Introduction

Retropharyngeal abscess (RPA) commonly occurs in children due to suppurative infection of retropharyngeal lymph nodes [1]. Adult RPA is unusual as these lymph nodes degenerate after the age of five years. Fever, neck pain and odynophagia or dysphagia should elicit the clinical suspicion of suppurative RPA. Due to its anatomical location, early recognition is essential to prevent serious local complications such as jugular necrotizing fasciitis, mediastinitis, empyema, aspiration pneumonia and airway obstruction [1]. If a retropharyngeal mass is associated with destructive lesions of the vertebrae, Pott’s disease (tuberculosis of the spine) with RPA should be suspected [2]. Pott’s disease occurs most commonly in the thoraco-lumbar region, followed by the cervical region [3]. Fever, night sweats, anorexia, and weight loss may not be present in tubercular RPA, therefore, it needs a high index of suspicion [4].

## Case presentation

**Figure 1:**
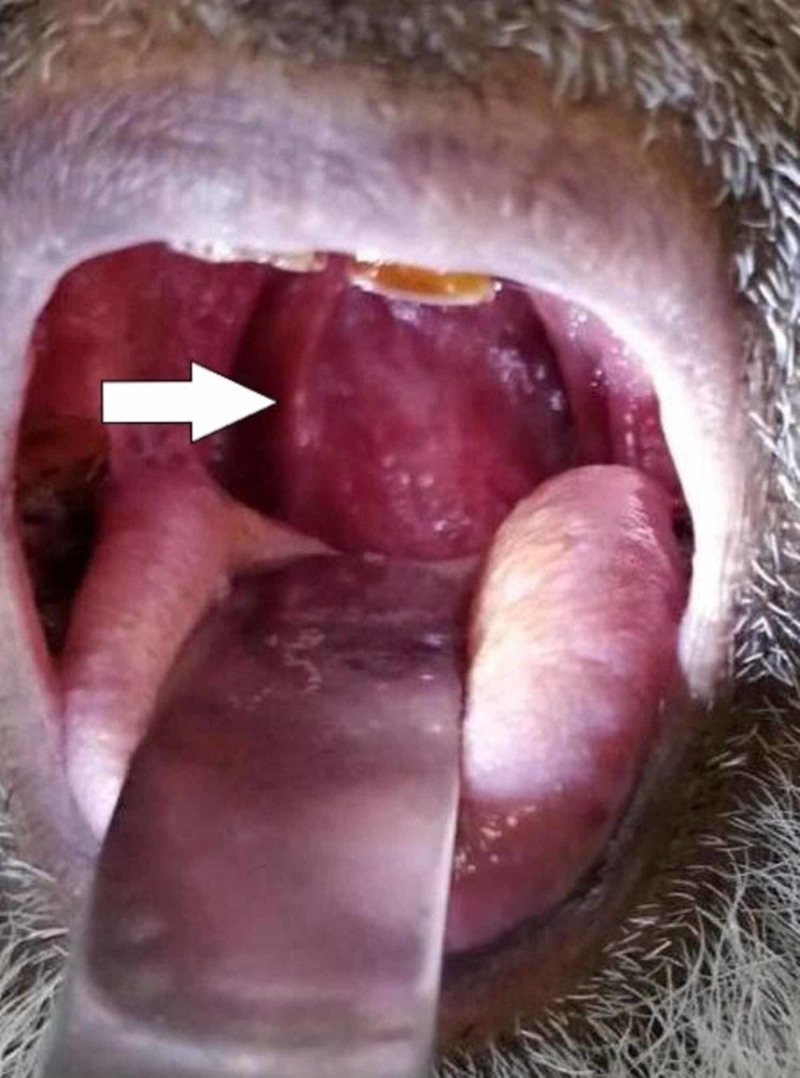
Examination of the oral cavity revealing a bulge (white arrow) on the posterior pharyngeal wall

A 70-year-old male presented to the outpatient department with a history of dysphagia and vague neck pain for three months, and breathing difficulty for three days. Dysphagia started with solid food, then it progressed to include liquids as well. Breathing difficulty was progressive and it increased on lying down. He had no fever, weight loss, cough, expectoration, or night sweats. There was no history of recurrent sore throat, dental pain, neck trauma, or neck instrumentation. There was no hoarseness, slurred speech, paresthesias or limb weakness. He did not have any associated co-morbidities. His vitals were normal and on neck examination, no palpable cervical lymph nodes, paraspinal muscle spasm or neck straightening was observed. Oropharyngeal examination revealed a bulge on the right posterior pharyngeal wall (Figure 1).

**Figure 2:**
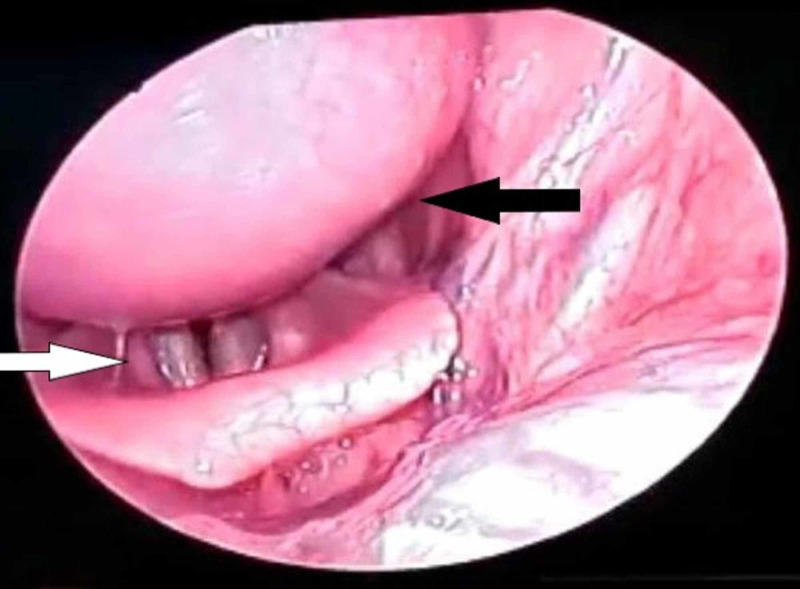
70° rigid endoscopy revealing the pharyngeal bulge (black arrow) extending till supraglottis (white arrow)

On 70° rigid endoscopy, the posterior pharyngeal wall bulge was seen extending down till supraglottis (Figure 2).

**Figure 3:**
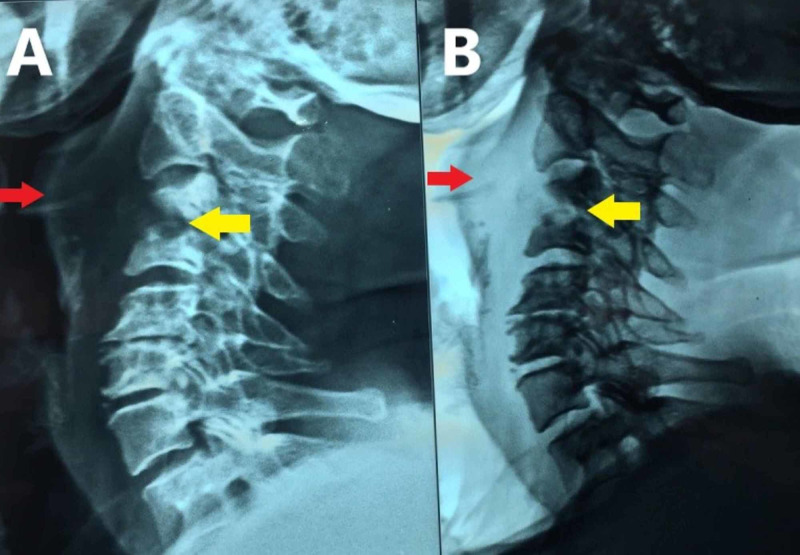
Lateral-view plain radiograph (A) and digital subtraction radiograph (B) revealing soft tissue mass (red arrows) in the prevertebral area displacing the airway anteriorly, and focal destruction (yellow arrows) of C3 and C4 vertebrae

A lateral-view neck radiograph revealed a soft tissue mass in the prevertebral area displacing the airway anteriorly, and focal destruction of C3 and C4 vertebrae (Figure 3).

A radiograph of chest and ultrasound of abdomen were essentially normal. The patient could not undergo a CT or MRI of the head, neck, and cervical spine due to financial constraints. Routine hematological and biochemical investigations were normal and erythrocyte sedimentation rate (ESR) was elevated at 70 mm in the first hour (normal range 0-20 mm).

**Figure 4:**
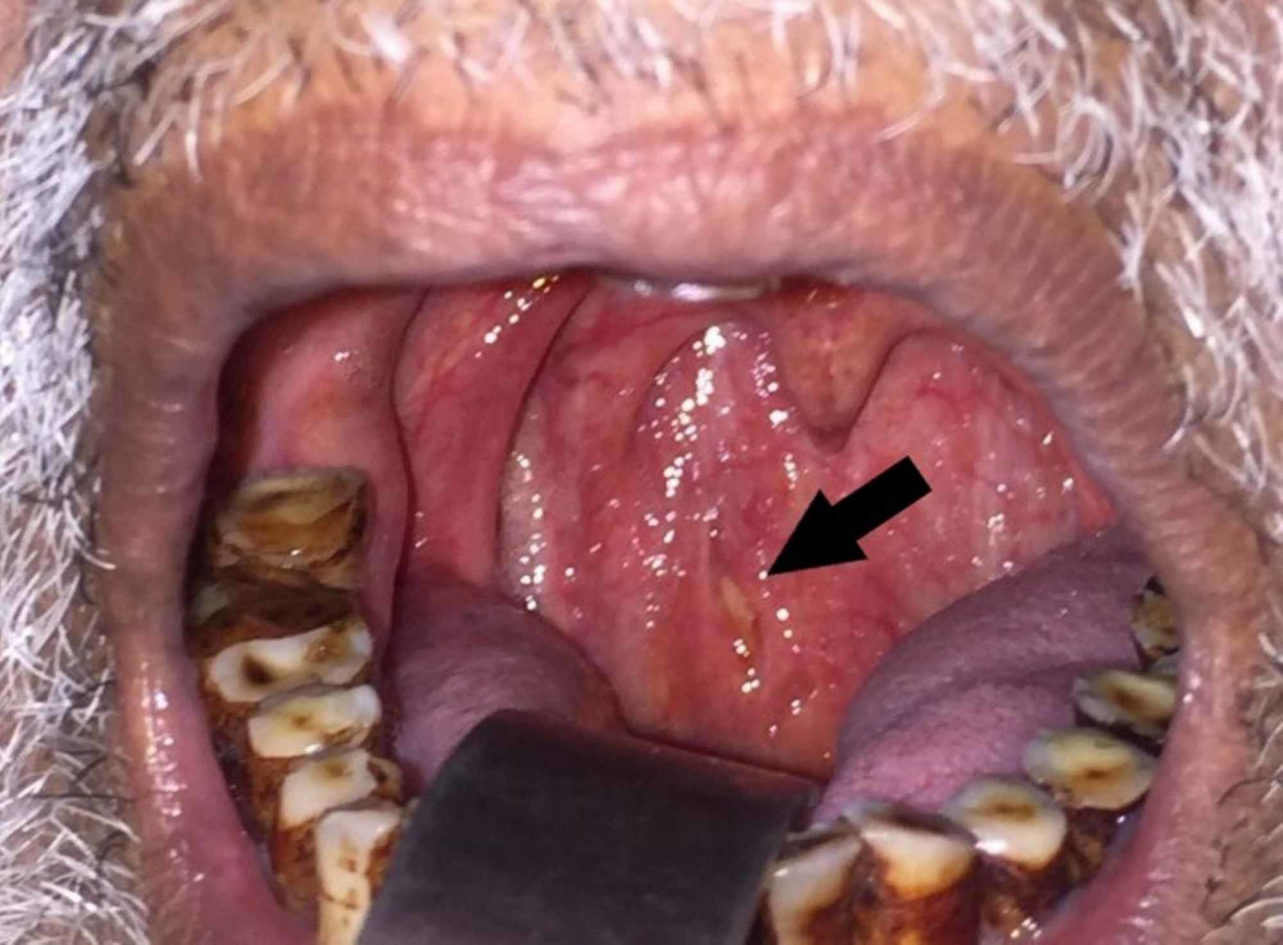
Examination of the oral cavity after drainage of pus. The site of incision has been marked with a black arrow

A provisional diagnosis of RPA with Pott’s disease was considered, and parenteral ceftriaxone, metronidazole and gentamicin were started. Written informed consent was obtained for an emergency tracheostomy, if so required. Incision and drainage of the abscess was performed under local anesthesia via oral route. A single vertical incision was given on the posterior pharyngeal wall, cutting the mucosa and pharyngeal constrictor muscle fibers, and pus was removed completely (Figure 4).

Regular suctioning in oropharynx using Yankauer tube was done to avoid aspiration. Gram stain of the pus was negative and the culture yielded no growth. Ziehl-Neelsen (ZN) stain revealed the presence of acid-fast bacilli. Subsequently, the pus sample tested positive for Mycobacterium tuberculosis by cartridge-based nucleic acid amplification test (CBNAAT). Treatment with isoniazid, rifampicin, pyrazinamide, and ethambutol was started. A cervical collar was advised for neck stabilization and the patient was discharged five days later.

## Discussion

The retropharyngeal space is a deep neck space surrounded by buccopharyngeal fascia anteriorly and alar fascial component of the prevertebral cervical fascia posteriorly [5]. It extends longitudinally downwards from the base of the skull to the posterior mediastinum. It contains lymph nodes that have drainage from the oropharynx, teeth and maxillary sinus, and they usually regress by the age of five years. In adults, acute retropharyngeal space abscess can be caused by penetrating trauma to the posterior mucosal wall of the pharynx and cervical oesophagus by a foreign body [6]. It is more common in immunocompromised patients, and endotracheal intubation and endoscopic procedures are usually implicated. Chronic RPA is usually of tuberculous etiology, originating from carious cervical spine or tuberculosis of retropharyngeal lymph nodes. The pathogen is carried hematogenously or lymphatically to the anterior and subchondral portions of the body of vertebrae, causing osteomyelitis, caseation necrosis, destruction of vertebrae, and prevertebral abscess or RPA formation.

The predominant symptoms are sore throat, fever, dysphagia, odynophagia, painful neck movements and difficulty in breathing [5]. On examination, posterior pharyngeal wall bulge, neck muscle spasm, palpable neck mass, drooling of saliva and stridor may be seen. Lateral cervical spine radiographs can display soft tissue widening, presence of air, osteolytic lesion in vertebrae and loss of cervical spine lordosis [4]. Lesions are better visualized on CT scan, which provides a three-dimensional view with higher resolution. MRI can better delineate soft tissues of the neck and assess vascular complications, such as internal jugular vein thrombosis.

RPA may cause significant airway compromise, which may necessitate tracheostomy. Endotracheal intubation may be unsafe, particularly with inexperienced hands, as it may lead to an inadvertent rupture of abscess into the unprotected airway and possible aspiration. Tracheostomy has been reported to be required in 3% to 22% of the patients [7]. Delayed treatment is associated with high morbidity and mortality due to complications such as airway obstruction, aspiration pneumonia, epidural abscess, erosion into the carotid artery, sepsis and jugular vein thrombosis [8]. RPA is a rare but well-recognized form of extra-pulmonary tuberculosis in the developing world [9-11]. It should be suspected in adults presenting with dysphagia and acute respiratory compromise [12]. The primary goal of the treating physician is to secure the airway at the earliest and then consider surgical drainage of RPA, followed by definitive anti-tubercular therapy.

## Conclusions

This report describes a clinical presentation with dysphagia and upper airway obstruction due to tubercular RPA associated with tuberculosis of the cervical spine. RPA is very rare in adults and occurs either in immunocompromised patients or in those who undergo instrumentation. In developing countries, tuberculosis needs to be considered as a differential in all cases of RPA.
